# Hippocampal subfield and amygdala nuclei volumes in schizophrenia patients with a history of violence

**DOI:** 10.1007/s00406-020-01098-y

**Published:** 2020-01-24

**Authors:** Natalia Tesli, Dennis van der Meer, Jaroslav Rokicki, Guttorm Storvestre, Cato Røsæg, Arvid Jensen, Gabriela Hjell, Christina Bell, Thomas Fischer-Vieler, Martin Tesli, Ole A. Andreassen, Ingrid Melle, Ingrid Agartz, Unn K. Haukvik

**Affiliations:** 1 Division of Mental Health and Addiction, Norwegian Centre for Mental Disorders Research (NORMENT), Oslo University Hospital, Nydalen, P.O. Box 4956, 0424 Oslo, Norway; 2 Norwegian Centre for Mental Disorders Research (NORMENT), Institute of Clinical Medicine, University of Oslo, Oslo, Norway; 3 School of Mental Health and Neuroscience, Faculty of Health, Medicine and Life Sciences, Maastricht University, Maastricht, The Netherlands; 4 Department of Psychology, University of Oslo, Oslo, Norway; 5 Department of Psychiatry, Oslo University Hospital, Oslo, Norway; 6 Department of Psychiatry, Ostfold Hospital Trust, Graalum, Norway; 7 Department of Mental Disorders, Norwegian Institute of Public Health, Oslo, Norway; 8 Department of Psychiatric Research, Diakonhjemmet Hospital, Oslo, Norway; 9 Department of Adult Psychiatry, Institute of Clinical Medicine, University of Oslo, Oslo, Norway; 10 Centre of Research and Education in Forensic Psychiatry, Oslo University Hospital, Oslo, Norway

**Keywords:** Aggression, Neuroimaging, MRI, Psychosis, Neuroanatomy, Hippocampus

## Abstract

Schizophrenia (SCZ) is associated with an increased risk of violence compared to the general population. Previous studies have indicated smaller hippocampal and amygdala volumes in violent than non-violent psychotic patients^.^ However, little is known about volumetric differences at the subdivision level of these structures. In the present study, hippocampal subfields and amygdala nuclei volumes were estimated with FreeSurfer from 3 T MRI of SCZ patients with (SCZ-V, *n* = 24) and without (SCZ-NV, *n* = 51) a history of severe violence and 90 healthy controls (HC). Volumetric differences between groups were explored with a general linear model covarying for confounders, in addition to follow-up analyses in patient groups controlling for clinical characteristics such as antipsychotic medication, duration of illness and illicit substance use. SCZ-V had smaller total hippocampal volume and smaller CA1, HATA, fimbria, and molecular layer of DG volumes compared to HC. Total amygdala volume together with basal nucleus, accessory basal nucleus, CTA, and paralaminar nucleus volumes were smaller in SCZ-V compared to HC. In SCZ-NV, compared to HC, the observed smaller volumes were limited to basal and paralaminar nucleus. There were no significant differences in hippocampal subfield and amygdala nuclei volumes between SCZ-V and SCZ-NV. Follow-up analyses showed that the results in patient groups were not affected by clinical characteristics. The results suggest that smaller hippocampal subfield and amygdala nuclei volumes may be relevant to violence risk in SCZ. However, the neurobiological signature of violence in SCZ should be further investigated in larger cohorts.

**Electronic supplementary material:**

The online version of this article (10.1007/s00406-020-01098-y) contains supplementary material, which is available to authorized users.

## Introduction

Violence in persons with schizophrenia (SCZ) constitutes a significant public health concern and contributes to the major stigma associated with mental illness. Epidemiological studies indicate that SCZ patients are at an increased risk of committing violent acts compared to the general population [1–4]. A large population-based study from Sweden estimated that one of ten male SCZ patients will be convicted for a violent offence within 5 years from the initial diagnosis [5]. Importantly, aggression and violence in SCZ are associated with different, though phenomenologically correlated factors including low socio-economic status, substance abuse and other psychopathological comorbidities [6]. Furthermore, aggression can be conceptualized in a dimensional manner on an axis where proactive and reactive forms coexist and influence each other [7]. Additionally, violent behavior in SCZ has been associated with exacerbating delusions during the states of acute psychosis [8, 9]. Bearing in mind the complexity of violence in SCZ, mapping its neurobiological signature represents an indispensable step towards improvement in therapeutic strategies and preventive measures.

Structural MRI (sMRI) studies of the brain consistently indicate SCZ to be associated with volume reductions in multiple cortical regions and subcortical structures, including the hippocampus and the amygdala [10–14]. It has been hypothesized that SCZ patients with a history of violence (SCZ-V) may be characterized by specific morphological brain abnormalities (e.g. lower volumes of prefrontal and temporal regions) distinguishing them from SCZ patients with no history of violence (SCZ-NV) [15, 16]. Smaller amygdala and hippocampal volumes have been associated not only with SCZ in general, but also with violence in SCZ in particular [17], emphasizing the role of these structures in emotional processing and impulse control. However, previous studies produced somewhat inconsistent results in SCZ-V, ranging from smaller hippocampal and/or amygdala volumes [18–20], no significant volumetric differences [21], to lower volume of hippocampus and increased volume of amygdala when compared to SCZ-NV [15].

Importantly, the hippocampus and the amygdala are not homogenous structures as both consist of morphologically differentiated subfields/nuclei subserving distinct functions. Owing to a tremendous progress in MRI data acquisition and analysis, it is now possible to interrogate them in vivo on a subdivision level [22–24]. The hippocampus is a C-shaped bilateral gray matter structure embedded in the temporal lobe. Histologically, it consists of the cornu ammonis (CA1–CA3), the dentate gyrus (DG, including a polymorphous CA4 layer), presubiculum, subiculum, and fimbria [25, 26]. Apart from its key role in learning, episodic and spatial memory, hippocampus is involved in a plethora of other behaviors and functions [27, 28]. Specifically, it plays a role in affect regulation and has been implicated in the processing of social emotions [29]. A recent meta-analysis [30] showed significant volume reductions in all investigated hippocampal subfields in SCZ when compared to HC. Furthermore, decreased volumes of CA1 have been associated with positive symptoms [31], whereas smaller subiculum volumes have been linked to negative symptoms [11]. The hypothetical involvement of specific hippocampal subfields in violence in psychotic offenders or associations between volumetric changes on the subfield level in relation to interpersonal aggression in psychiatric populations have not yet been addressed.

In contrast, more attention has been given to the unique relationship between the anatomical subdivisions of the amygdala and aggression. This almond-shaped structure is anatomically divided into three nuclear complexes including the basolateral complex (lateral, basal, and accessory basal nuclei), the central complex (central and medial nuclei) and the superficial nuclei (including cortico-amygdaloid transition zone) [32, 33]. The amygdaloid complex receives multimodal inputs and plays a pivotal role in both integration of motivationally salient stimuli and subsequent transmission of this information to a wide range of cortical and subcortical regions [34]. The lateral nucleus of the basolateral complex is viewed as a sensory input gateway, whereas the central nucleus is thought to serve the output role for innate emotional responses [7].

Evidence from studies on rodents and non-human primates indicates that the nuclei of the amygdala play different roles in aggression, with central and medial amygdala being particularly important for aggression [35–37]. Additionally, human studies showed that the dorsal (central complex) and the ventral (basolateral complex) components of the amygdala are volumetrically differently associated with aggression and impulsivity in psychiatric populations [38] and with reactive aggression in non-psychotic populations [39].

As outlined above, the overwhelming majority of sMRI studies investigated the role of the hippocampus and the amygdala in aggression by treating these structures as homogenous, without further parcellation. To our best knowledge, there have been no previous studies mapping volumetric changes in the hippocampus and the amygdala at the subdivision level in SCZ-V. Such a detailed interrogation may bring us closer to elucidating neurobiological mechanisms underpinning violence in SCZ.

In the current study, we applied a robust, automated parcellation method with a high level of segmentation accuracy [23, 24] to identify neurobiological correlates of trait violence (i.e. history of severe violent offending) in SCZ by simultaneously measuring volumes in hippocampal subfields and nuclei of amygdala. We hypothesized that both SCZ groups would have global volume reductions in the hippocampus and the amygdala compared to HC, and that SCZ-V would have more pronounced volumetric decreases on the subfield and nuclei level than SCZ-NV. Based on the previous findings on aggression and amygdala subdivisions, we hypothesized the nuclei of the basolateral complex as well as central nuclei to be the most affected. Due to paucity of research on hippocampal subdivisions and aggression in general and in SZ, our investigation had an exploratory character aiming at a better understanding of this heterogenous structure in relation to violence in SCZ.

## Methods

### Sample

The subject sample (*n* = 165) consisted of SCZ-V (*n* = 24), SCZ-NV (*n* = 51), and HC (*n* = 90). The participants from SCZ-V group were recruited from high-security forensic psychiatric wards at Østfold Hospital and Oslo University Hospital, Norway. The SCZ-V group was comprised of patients with a Diagnostic and Statistical Manual of Mental Disorders (DSM-IV) diagnosis of paranoid SCZ (DSM-IV 295.3, *n* = 19), undifferentiated SCZ (DSM-IV 295.9, *n* = 2) or residual SCZ (DSM-IV 295.6, *n* = 3), male/female ratio 23/1. Inclusion criteria for this group were in addition to diagnosis, also history of murder or attempted murder as well as severe physical assault towards other people (including sexual assaults) according to the MacArthur criteria [40]. The participants from the SCZ-NV group were recruited from four major psychiatric hospitals and their affiliated outpatient clinics that cover most of the population in Oslo, Norway. The SCZ-NV group consisted of sex-matched SCZ patients without previous history of interpersonal violence and with a corresponding diagnostic profile, that is paranoid SCZ (DSM-IV 295.3, *n* = 40), undifferentiated SCZ (DSM-IV 295.9, *n* = 6), residual SCZ (DSM-IV 295.6, *n* = 4), and disorganized SCZ (DSM-IV 295.1, *n* = 1), male/female ratio 50/1. The HC cohort (*n* = 90) matched for sex (male/female ratio 87/3) and within the same age range as SCZ patients was extracted from a larger HC group randomly selected from the national records. All participants in the study were obtained from the on-going multi-center TOP (thematically organized psychosis) study in Oslo, Norway. The inclusion criteria were age between 18 and 65 years, no head trauma leading to loss of consciousness, and the absence of previous or current somatic illness that might affect brain morphology. Some of the SCZ-NV and HC subjects may have been included in the previous large MRI meta-analyses (comprising over 4000 subjects) in the ENIGMA studies of whole hippocampal volumes in schizophrenia [12] and bipolar disorders [41].

The study was approved by the Norwegian Regional Committee for Medical Research Ethics and the Norwegian Data Inspectorate. Written informed consent was obtained from all participants in the study.

### Clinical assessment

All patients were thoroughly assessed by trained psychologists and physicians. Clinical diagnoses were confirmed with the Structured Clinical Interview for DSM-IV axis I disorders (SCID-I) [42]. The SCZ-V had diagnostic evaluation based on detailed medical records as well as forensic reports. The diagnoses of SCZ-NV were confirmed by clinical interviews as well as supplementary information drawn from medical records. Psychosocial functioning was evaluated with the Global Assessment of Function (GAF) scale (split version). Affective symptoms were assessed with the Young Mania Rating Scale (YMRS) and the Calgary Depression Scale for Schizophrenia (CDSS). Alcohol and illicit substance use were evaluated with The Alcohol Use Disorders Identification Test (AUDIT) and The Drug Use Disorders Identification Test (DUDIT), respectively. Current psychotic symptoms were rated using the Positive and Negative Syndrome Scale (PANSS) [43].

The SCZ samples included medicated (*n* = 69), unmedicated (*n* = 4) patients as well as patients with missing medication status (*n* = 2). Defined daily dosages (DDD) of current antipsychotic medication use were calculated in line with the guidelines from WHO (https://www.whocc.no/atc_ddd_index/).

The medical charts of SCZ-NV patients were carefully inspected to confirm/disconfirm absence of previous history of violence. This procedure encompassed thorough evaluation of all study inclusion protocols which are based on comprehensive information obtained from medical records, including data from clinical journals and detailed interview with the patient. All patients in the SCZ-NV group who had scores above 4 on item G14 (poor impulse control, PANSS) were excluded from the study.

HC subjects were screened with the Primary Care Evaluation of Mental Disorders (Prime-MD) questionnaire [44] and interviewed by trained clinical psychologists to confirm no history of psychiatric disorder.

### MRI acquisition and processing

MRI data were acquired using two GE 3 T scanners due to a hardware upgrade. The MRI data obtained before the upgrade were collected on a 3 T GE Signa HDxt scanner (GE Medical Systems, Milwaukee, WI, USA) using a standard 8-channel head coil at Oslo University Hospital, Norway. T1-weighted volumes were acquired using a sagittal 3D fast spoiled gradient echo (FSPGR) sequence with the following parameters: repetition time (TR) 7.8 ms, echo time (TE) 2.9 ms, flip angle 12°, slice thickness 1.2 mm, 166 slices, field of view (FOV) 256 mm × 256 mm, acquisition matrix 256 × 192 mm, reconstructed in-plane resolution 256 × 256 mm/pix. MRI data after the upgrade were collected on a 3 T GE 750 Discovery scanner using a 32-channel head coil at Oslo University Hospital. T1-weighted volumes were acquired using a sagittal 3D BRAVO sequence with the following parameters: repetition time (TR) 8.2 ms, echo time (TE) 3.2 ms, flip angle 12°, slice thickness 1.0 mm, 192 slices, field of view (FOV) 256 mm × 256 mm. All MRI scans were evaluated by a neuroradiologist to ensure no brain pathology affecting the analyses.

**Fig. 1:**
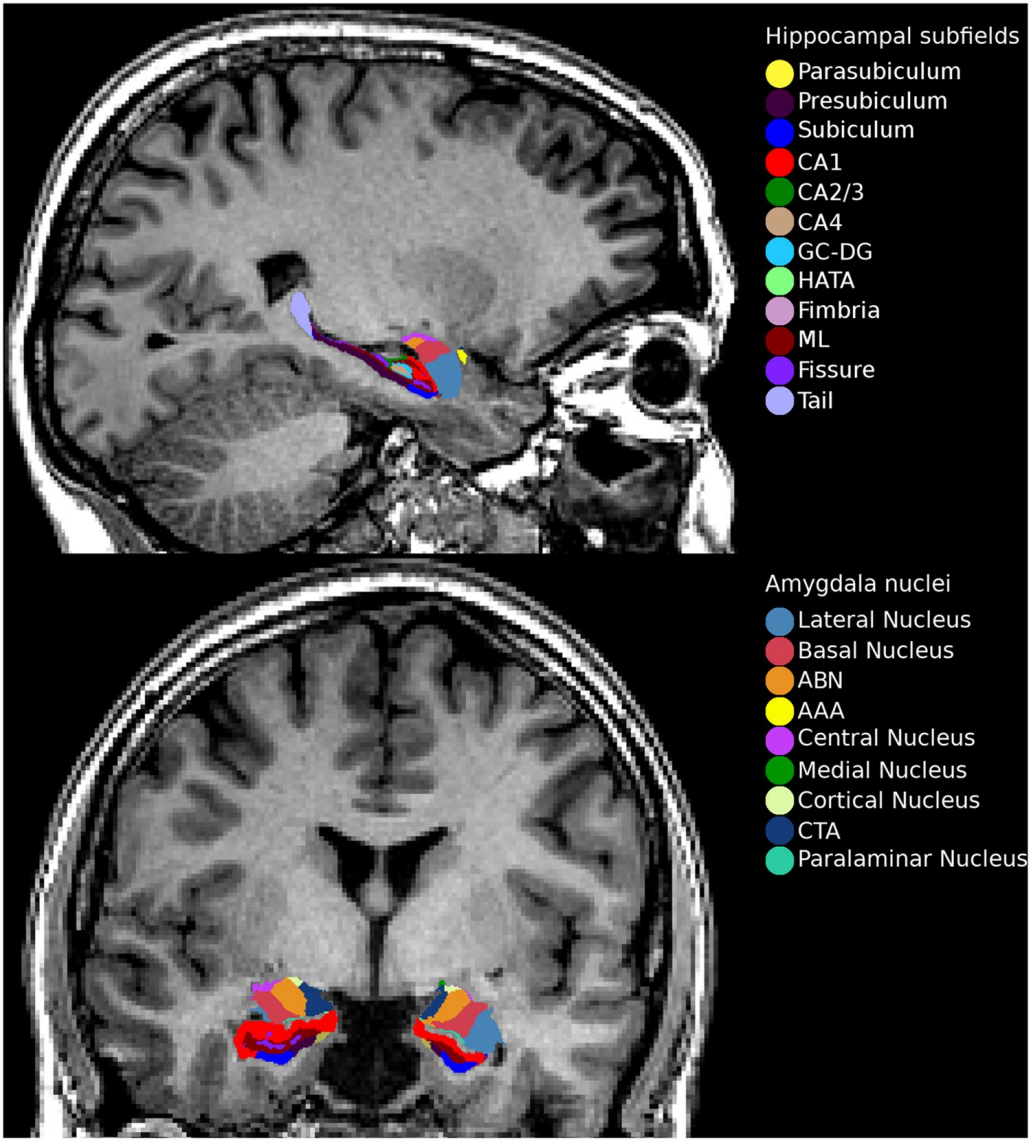
Sagittal and coronal view of the FreeSurfer 6.0 hippocampal subfield and amygdala nucleus segmentation. *CA* cornu ammonis, *GC-DG* granule cell layer of dentate gyrus, *HATA* hippocampal–amygdaloid transition area, *ML* molecular layer, *ABN* accessory basal nucleus, *AAA* anterior amygdaloid area, *CTA* cortico-amygdaloid transition area

Briefly, T1-weighted MRI volumes were pre-processed using the standard FreeSurfer recon-all pipeline (version 5.3) (https://surfer.nmr.mgh.harvard.edu/). Hippocampal subfield and amygdala nuclei volume estimates were subsequently obtained by applying the hippocampal subfield segmentation algorithm released by FreeSurfer (version 6.0). This tool employs a probabilistic atlas based on Bayesian inference and created with ultra-high resolution ex vivo MRI data (~ 0.1–0.15 mm isotropic) to generate an automated segmentation of the hippocampus and the amygdala. Simultaneous parcellation of both structures ensures that they do not overlap and excludes the possibility that there are gaps between them [23, 24]. Hippocampal segmentation included 12 subfields: the parasubiculum, presubiculum, subiculum, cornu ammonis fields 1, 2/3, and 4 (henceforth referred to as CA1, CA3, and CA4), granule cell layer of dentate gyrus (DG), hippocampal–amygdaloid transition area (HATA), fimbria (a white matter structure), the molecular layer of DG, hippocampal fissure, and the hippocampal tail. Nuclei of the amygdala included nine subdivisions: the lateral, basal and accessory basal, central, medial, cortical and paralaminar nucleus, the anterior amygdaloid area as well as the cortico-amygdaloid transition area (CTA) (Fig. 1).

### Statistical analyses

#### Demographics and clinical characteristics

Descriptive statistical analyses were performed using R (version 3.5.3, www.R-project.org). The analysis of variance, Person’s Chi-squared or *t *test were applied to assess group differences on age, sex as well as psychometric measures, use of medication, alcohol and illicit substance use. All statistical tests were two tailed with statistical significance reported at the 0.05 level.

#### Volumetric analyses

All statistical analyses of volumetric MRI data were performed with R (version 3.5.3, www.R-project.org). 12 hippocampal subfields and 9 nuclei of the amygdala were included in the subsequent analyses. Whole hippocampal volume was defined as the sum of all subfields minus the hippocampal fissure whereas whole amygdala volume was defined as the sum of all nuclei. The estimates of both hemispheres were summed together to reduce the number of analyses thus minimizing the multiple testing burden and increasing statistical power. Volumes of whole hippocampus and whole amygdala were checked for outliers in all subjects and discarded from the analyses if larger than four standard deviations from the mean.

#### Primary analyses

Main effects of diagnostic group (SCZ-V, SCZ-NV and HC) on volumes (whole hippocampus together with 12 hippocampal subfields as well as whole amygdala together with 9 amygdala nuclei) were tested using a general linear model (GLM) by creating 3 pairwise contrasts (SCZ-NV versus HC, SCZ-V versus HC, SCZ-V versus SCZ-NV) covarying for age, age^2^, sex, intracranial volume (ICV), and scanning site. Effect sizes were calculated with Cohen’s *d*. All *p *values were adjusted for multiple comparisons with false discovery rate (FDR) [45].

#### Secondary analyses

We repeated our analyses in patient groups (SCZ-V and SCZ-NV) using the same GLM as in the primary analysis and covarying for duration of illness, illicit substance use (DUDIT), and antipsychotic medication use (DDD) in three separate tests. All *p *values were adjusted for multiple comparisons with false discovery rate (FDR) [45].

## Results

### Clinical and demographic characteristics

Clinical and demographic statistics are summarized in Table 1. There was a significant main effect of group on age (*F*_2,162_ = 6.12, *p* = 0.002), education (*F*_2,156_ = 22.36, *p* = 0.001), and illicit substance abuse (*F*_2,147_ = 28.8, *p* = 0.001), with higher age in SCZ-V and HC compared to SCZ-NV, longer education in HC compared to both SCZ groups (HC > SCZ-NV > SCZ-V) and higher illicit substance use in SCZ-V compared to SCZ-NV and HC (SCZ-V > SCZ-NV > HC). Additionally, SCZ-V had a significantly lower age at psychosis onset (*t*_41_ = 2.63, *p* = 0.011), lower age at first admission for psychosis (*t*_39_ = 2.64, *p* = 0.011) and longer duration of illness (*t*_31_ = − 3.51, *p* = 0.001) compared to SCZ-NV. There was also a significant difference in CDSS with SCZ-NV scoring higher compared to SCZ-V (*t*_45_ = 3.13, *p* = 0.002). Finally, there was a trend-significant difference in antipsychotic medication use between the patient groups with SCZ-NV having higher DDD than SCZ-V (*t*_29_ = -1.95, *p* = 0.06). There were no other significant differences on a group level for other demographic or clinical variables.

**Table 1:** Demographic variables and clinical characteristics

	SCZ-V (n = 24)		SCZ-NV (n = 51)		HC (n = 90)		Chi-square
	*n*	*n *(%)	*n*	*n* (%)	*n*	*n* (%)	
Sex (m/f)	23/1	96/4	50/1	98/2	87/3	97/3	*p* = 0.847
Cannabis, last 2 weeks (no/yes)^a^ (*n* = 24/50)	22/2	91/9	44/6	88/12			*p* = 0.939
Cannabis, last 2 years (no/yes)^a^ (*n* = 22/50)	11/11	50/50	22/28	44/56			*p* = 0.830

	Mean (SD)	Range	Mean (SD)	Range	Mean (SD)	Range	*t *test
Alcohol last 2 weeks (units)^a^ (*n* = 22/49)	1.59 (4.1)	0–18	5.0(12.52)	0–72			*p* = 0.094
Alcohol last 2 years (units)^a^ (*n* = 21/46)	372.86 (731.72)	0–2496	511.24(878.46)	0–4160			*p* = 0.504
CDSS^a^ (*n* = 18/49)	1.89 (2.47)	0–7	4.35(3.67)	0–15			***p* = 0.002**
GAF symptom^a^ (*n* = 21/51)	43.33 (13.98)	28–73	44.71(15.15)	21–91			*p* = 0.718
GAF function^a^ (*n* = 21/51)	40.38 (15.19)	20–78	45.8(14.25)	21–85			*p* = 0.169
PANSS positive^a^ (*n* = 20/51)	16.65 (7.36)	7–28	14.67 (5.61)	7–32			*p* = 0.286
PANSS negative^a^ *(*n* = 21/51)	18.62 (6.62)	8–33	17.61 (6.47)	7–43			*p* = 0.556
PANSS general^a^ (*n* = 20/51)	29.85 (9.9)	18–49	32.61 (8.6)	17–69			*p* = 0.282
Age at psychosis onset^a^ (*n* = 23/50)	19.04 (5.38)	10–30	22.56 (5.13)	14–39			***p* = 0.011**
Age at first psychosis admission^a^ (*n* = 19/40)	20.68 (4.92)	10–29	24.48 (5.57)	15–41			***p* = 0.011**
Duration of illness^a^ (*n* = 22/49)	13.6 (8.53)	3.06–28.93	6.53 (6.02)	0.35–22.15			***p* = 0.001**
Antipsychotics (DDD)^a^ (*n* = 20/48)	1.81 (1.07)	0.47–4.09	1.28 (0.85)	0.05–5			*p* = 0.06

	Mean (SD)	Range	Mean (SD)	Range	Mean (SD)	Range	ANOVA
Age (years)^a^ (*n* = 24/51/90)	33.84 (8.21)	19.2–49.1	28.89 (6.95)	18.76–48.86	33.18 (7.72)	19.45–46.22	***p* = 0.002**
Years of education^a^ (*n* = 22/51/86)	10.7 (1.82)	9–15	13.45 (2.8)	4.5–20	14.53 (2.29)	11–25	***p* < 0.000**
AUDIT^a^ (*n* = 19/48/83)	5.21 (6.09)	0–22	5.83 (5.83)	0–22	5.39 (3.01)	0–13	*p* = 0.822
DUDIT^a^ (*n* = 18/49/82)	8.44 (9.9)	0–29	4.39 (6.53)	0–31	0.09 (0.36)	0–2	***p* < 0.000**

*SCZ-V* schizophrenia patients with a history of violence, *SCZ-NV* schizophrenia patients with no history of violence, *HC* healthy controls, m/f male/female, *SD* standard deviation, *PANSS *Positive and Negative Syndrome Scale, *CDSS* Calgary Depression Scale for Schizophrenia, *GAF* Global Assessment of Function split version, *DDD* defined daily dosage, *AUDIT* alcohol use disorder identification test, *DUDIT* drug use disorder identification test

Bold *p* value indicates significant differences between groups

^a^Valid scores in brackets

### Volumetric analyses

#### Primary analyses

**Fig. 2:**
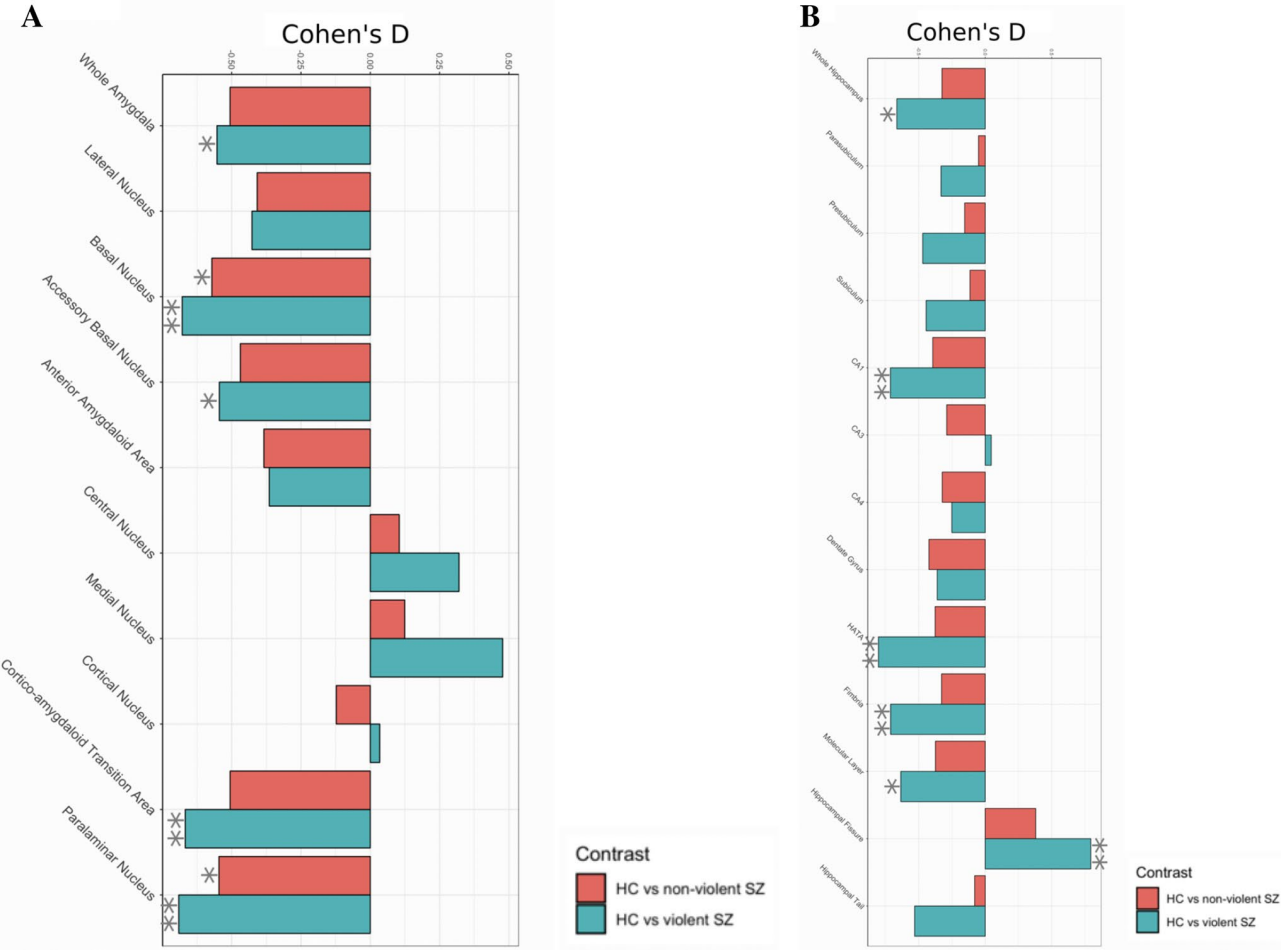
Hippocampal subfield and amygdala nuclei volume differences between SCZ-V, SCZ-NV and HC. Visualization of effect sizes calculated with Cohen’s *d*. **a** Volumetric differences in hippocampal subfields between SCZ-V versus HC and SCZ-NV versus HC. **b** Volumetric differences in amygdala nuclei between SCZ-V versus HC and SCZ-NV versus HC. Results corrected for age, age^2^, sex, intracranial volume (ICV), and scanning site. *SCZ-V* schizophrenia patients with a history of violence, *SCZ-NV* schizophrenia patients with no history of violence, *HC* healthy controls, *CA* cornu ammonis, *HATA* hippocampal–amygdaloid transition area. *Significant *p *values < 0.05 (FDR-corrected). **Significant *p *values <0.01 (FDR corrected)

The results from the primary volumetric analyses are summarized in Table 2 and visualized in Fig. 2. Violin plots showing distributions of the original brain volumes are shown in supplementary Fig. 1.

**Table 2:** Results from regression analysis of hippocampal subfield and amygdala nuclei volumes in SCZ-V, SCZ-NV, and HC

Region	SCZ-NV vs HC		SCZ-V vs HC		SCZ-V vs SCZ-NV	
	*p *value	Effect size	*p* value	Effect size	*p* value	Effect size
Whole hippocampus	0.144	− 0.325	**0.02**	− 0.664	0.503	− 0.279
Parasubiculum	0.789	− 0.05	0.168	− 0.332	0.503	− 0.324
Presubiculum	0.575	− 0.154	0.061	− 0.47	0.503	− 0.344
Subiculum	0.64	− 0.114	0.075	− 0.444	0.503	− 0.34
CA1	0.097	− 0.394	**0.009**	− 0.712	0.503	− 0.289
CA3	0.176	− 0.289	0.879	0.045	0.503	0.311
CA4	0.144	− 0.323	0.332	− 0.251	0.881	0.063
GCMLDG	0.097	− 0.423	0.17	− 0.361	0.881	0.055
HATA	0.097	− 0.376	**0.009**	− 0.803	0.503	− 0.419
Fimbria	0.136	− 0.328	**0.009**	− 0.711	0.503	− 0.396
Molecular layer	0.097	− 0.374	**0.025**	− 0.635	0.654	− 0.213
Hippocampal fissure	0.097	0.379	**0.009**	0.793	0.503	0.384
Hippocampal tail	0.691	− 0.079	0.067	− 0.53	0.503	− 0.361
Whole amygdala	0.051	− 0.505	**0.033**	− 0.553	0.881	− 0.072
Lateral nucleus	0.097	− 0.407	0.102	− 0.426	0.957	− 0.025
Basal nucleus	**0.046**	− 0.571	**0.009**	− 0.678	0.81	− 0.133
Accessory basal nucleus	0.064	− 0.468	**0.033**	− 0.544	0.861	− 0.11
Anterior amygdaloid area	0.097	− 0.383	0.163	− 0.364	0.987	0.003
Central nucleus	0.644	0.104	0.179	0.32	0.654	0.224
Medial nucleus	0.617	0.124	0.061	0.477	0.503	0.386
Cortical nucleus	0.617	− 0.122	0.879	0.034	0.776	0.165
Corticoamygdaloid transition area	0.051	− 0.505	**0.009**	− 0.667	0.69	− 0.215
Paralaminar nucleus	**0.046**	− 0.545	**0.009**	− 0.69	0.776	− 0.15

Effects of diagnostic group (SCZ-V, SCZ-NV, and HC) on hippocampal subfield and amygdala volumes were tested using general linear model by creating three pairwise contrasts (SCZ-NV versus HC, SCZ-V versus HC, SCZ-V versus SCZ-NV) covarying for age, age^2^, sex, intracranial volume (ICV), and scanning site. All *p *values are FDR corrected for multiple comparisons. Bold *p *values indicate significant differences between groups. Effect sizes are calculated with Cohen´s D

*SCZ-V* schizophrenia patients with a history of violence, *SCZ-NV* schizophrenia patients with no history of violence, *HC* healthy controls, *CA* cornu ammonis, *GCMLDG* granule cell layer of the dentate gyrus, *HATA* hippocampal–amygdaloid transition area

Pairwise comparisons showed that whole hippocampal volume was significantly smaller in SCZ-V compared to HC (*p* = 0.02, *d* = − 0.664). On the subfield level, we found that CA1 (*p* = 0.009, *d* = − 0.712), HATA (*p* = 0.009, *d* = − 0.803), fimbria (*p* = 0.009, *d* = − 0.711), and molecular layer (*p* = 0.025, *d* = − 0.635) were significantly smaller, whereas hippocampal fissure (*p* = 0.009, *d* = 0.793) was significantly larger in SCZ-V compared to HC. There were no significant volumetric differences for whole hippocampus/hippocampal subfields for other pairwise comparisons.

Whole amygdala was significantly smaller in SCZ-V when compared to HC (*p* = 0.033, *d* = − 0.553). On the subdivision level, both SCZ-V and SCZ-NV had significant volumetric decreases in basal nucleus (SCZ-V: *p* = 0.009, *d* = − 0.544; SCZ-NV: *p* = 0.046, *d* = − 0.571) and paralaminar nucleus (SCZ-V: *p* = 0.009, *d* = − 0.69; SCZ-NV: *p* = 0.046, *d* = − 0.545) compared to HC. Additionally, accessory basal nucleus (*p* = 0.033, *d* = − 0.544) and CTA (*p* = 0.009, *d* = − 0.667) were significantly smaller only in SCZ-V group compared to HC. There were no significant volumetric differences in whole amygdala/amygdala nuclei for other pairwise comparisons. However, there was a clear step-wise pattern of larger effect sizes for all subfields and nuclei in SCZ-V compared to HC than in SCZ-NV and HC, as can be seen in Fig. 2, indicating more pronounced volumetric abnormalities in SCZ-V group.

#### Secondary analyses

There were no significant volumetric differences on hippocampal subfields or amygdala nuclei between SCZ-V and SCZ-NV after controlling for illicit substance use (DUDIT), duration of illness or antipsychotic medication (DDD).

These results are summarized in Table 1 in supplementary materials.

## Discussion

Our results revealed significantly smaller total hippocampal and amygdala volumes as well as smaller hippocampal subfields and amygdala nuclei in SCZ-V when compared to HC. In SCZ-NV the observed smaller volumes were limited to several amygdala nuclei when compared to HC. This is to the best of our knowledge the first study assessing associations between hippocampal subfields, amygdala nuclei volumes, and violence in SCZ.

The smaller total hippocampal and amygdala volumes in SCZ-V are in line with previous studies (see [17] for review). Regarding specific amygdala nuclei analyses, the SCZ-V but not the SCZ-NV group showed smaller accessory basal nucleus and CTA compared to HC. The accessory basal nucleus has extensive internuclear connections with the basal nucleus and is one of the main targets for inputs from cortical as well as subcortical regions [32]. Although both SCZ groups showed smaller volumes of the basal and paralaminar nucleus compared to HC, the effect sizes were greater for the SCZ-V group. The basal nucleus belongs to the basolateral complex and connects to striatal areas implicated in control of instrumental behaviours [46] and in generating emotional states via signalling affective arousal to higher-order brain areas [47]. Additionally, this nucleus is the main target of afferents from the prefrontal cortex [32]. The anatomical and functional connectivity between the orbital prefrontal cortex and the basal amygdala are essential for decoding emotionally vital information and thus are critical for guiding goal-directed behaviours [48, 49]. The paralaminar nucleus, on the other hand, being closely associated with the basal nucleus projects to the central nucleus as well as to the ventral striatum and receives afferent inputs from the rostral CA1 and subiculum [50]. The anatomical circuit involving hippocampal inputs to paralaminar nucleus is speculated to be involved in contextual fear learning [51]. It has been argued that psychotic and impulsive aggression is characterized by excessive fear conditioning, while predatory (psychopathic) aggression is linked to deficient fear conditioning [52]. A previous study by Gopal et al. [38] showed volumes of the ventral region of the amygdala (corresponding roughly to the basolateral complex) to be bilaterally positively correlated with motor impulsivity in a mixed population of psychiatric patients. A similar study in a non-psychiatric population found a negative correlation between reactive aggression and volumes of lateralized ventral amygdala [39]. These disparate results do not render a straightforward comparison to our findings due to stark methodological differences in the amygdala parcellation procedure and sample characteristics. Still, the observed volumetric decreases in the amygdala are in line with our hypothesis of more pronounced differences on the segmentation level in the SCZ-V than in the SCZ-NV group.

Previous research indicates a substantial co-morbidity between SCZ and psychopathy in forensic populations [53]. Furthermore, it has been shown that amygdala is differently affected in a subgroup of psychopathic individuals characterized by callous–unemotional (CU) personality traits [54, 55]. High scores on CU facet of psychopathy have been associated with amygdala hypo-reactivity to negatively valanced emotional stimuli such as fearful facial expressions [56], whereas structural MRI studies have shown mixed effects, with the majority of findings indicating smaller volumes of amygdala compared to HC [57, 58], albeit segmentation studies investigating regional abnormalities of amygdala in this subgroup are still scarce [59]. As the participants in the SCZ-V group were recruited from high-security forensic psychiatry wards and convicted for a violent crime, it is possible that these acts of violence were committed in a state of psychosis and as such driven by paranoid delusions and/or command hallucinations. It has also been estimated that over 50% of SCZ patients who had committed homicide did experience exacerbating delusions in the period antecedent to the act of homicide [8]. However, we cannot exclude the possibility that violence in our SCZ-V group was confounded by possible co-morbidity with psychopathic traits. Bearing in mind that particular patterns of amygdala dysfunction have been shown to be contingent upon particular etiopathogenetic types of aggression and violence, we can only stipulate that psychopathic traits might have affected our results, as SCZ-V participants were not screened for these characteristics.

The hippocampal subfield analyses showed significant volumetric decreases in CA1, HATA, fimbria, and molecular layer in SCZ-V compared to HC. The observed large effect sizes for CA1 volumetric reductions are in line with our recent meta-analysis [30], indicating this region to be the most affected in SCZ in general. Additionally, CA1 has been shown to be selectively decreased in the early stages of SCZ [60] with its subsequent volumetric decline being correlated with increased symptom severity over time [61]. Indeed, it has been reported that volumetric deficits in CA1 reflect symptom severity and are linked to the amount of antipsychotic medication required to control these symptoms [62]. As reductions in CA1 are thought to be involved in the neurobiological mechanisms of delusions and hallucinations, we may speculate that these reductions could be relevant to violence risk in SCZ.

However, since the smaller hippocampal subfield volumes were only present in the SCZ-V and not in the SCZ-NV group compared to HC, even though the SCZ-NV was twice the size the SCZ-V group, we might speculate that the smaller volumes in SCZ-V could reflect an overall higher symptomatic burden associated with SCZ rather than violence history. Moreover, we found smaller global hippocampus and amygdala volumes, in line with the largest meta-analysis on subcortical volumes in SCZ to date [12], but these were also limited to the SCZ-V group. The SCZ-V patients had longer duration of illness and higher use of antipsychotic medication than the SCZ-NV group, both of which may have an impact on brain morphology [63–65]. However, the subsequent secondary analyses in SCZ groups indicated that the volumetric differences between the patient groups were not confounded by duration of illness, antipsychotic medication use or illicit substance use. The lack of significant differences between SCZ-NV and SCZ-V together with larger effect sizes in SCZ-V compared to HC than in SCZ-NV compared to HC are also in line with the results from our previous study of white matter microstructure in this group [66]. Taken together, the results from both studies emphasize the difficulties of disentangling effects of illness severity from violence or aggression traits on brain morphology within this patient group.

The present study has some limitations. First, the sample size in the SCZ-V group was relatively small. Thus, it is possible that the lack of significant differences between patient groups could be due to type II errors, as we found an overall pattern of larger effect sizes in SCZ-V compared to HC than in SCZ-NV compared to HC. However, the size of our SCZ-V sample matches previous studies investigating volumetric correlates of violence and aggression in SCZ-V (*n* = 10–37) [19–21]. Moreover, the only study investigating associations between volumes of amygdala subdivisions and aggression in psychiatric populations ([38], *n* = 41) included nine SCZ patients, the remaining individuals having a wide spectrum of diagnoses including bipolar disorder and ADHD. There was also one scanner upgrade during the inclusion of study participants. We accounted for this by splitting and matching subject cohorts before and after scanner upgrade as well as including scanner as a covariate in the statistical analyses. Additionally, we used FDR procedure to control for multiple comparisons. This method is considered less conservative than FWER procedures, thus our results should be interpreted with caution.

Our study has several strengths. We applied a robust automated hippocampal and amygdala segmentation algorithm (FreeSurfer, v6.0) which allowed a thorough investigation of these two structures simultaneously. Additionally, violence in the SCZ-V group was operationalized according to the MacArthur criteria with a stringent inclusion protocol comprising exclusively individuals who committed serious acts of violence (murder, attempted murder as well as severe physical assaults towards other people).To ensure a high level of clinical homogeneity, the patient groups included only participants with a SCZ diagnosis and no other psychotic disorders.

## Conclusions and future directions

In summary, our results revealed a pattern of smaller volumes in several hippocampal subfields and amygdala nuclei of importance to emotion regulation, control of instrumental behaviour, and fear conditioning in SCZ patients with a history of severe interpersonal violence compared to HC. We found no significant differences between the SCZ groups and hence no specific volumetric correlates of trait violence in SCZ despite the larger effect sizes in SCZ-V. The neurobiological signature of violence in SCZ should be further investigated by increasing the sample size. Further, to disentangle brain volumetric abnormalities related to illness severity from those related to antisocial traits, a non-psychotic violence cohort should be included in future analyses.

## Electronic supplementary material

Below is the link to the electronic supplementary material.

Supplementary file1 (PDF 126 kb)

Supplementary file2 (PDF 255 kb)
