# Supplementary material for: Hippocampal subfield and amygdala nuclei volumes in schizophrenia patients with a history of violence
Source: Eur Arch Psychiatry Clin Neurosci. 2020 Jan 24;270(6):771–82. doi: 10.1007/s00406-020-01098-y (PMC7423802; doi:10.1007/s00406-020-01098-y)
Supplement: Supplementary file 1 — Supplementary file1 (PDF 126 kb) [file 406_2020_1098_MOESM1_ESM.pdf]

Supplementary Table 1 Results from regression analyses on hippocampal subfields and amygdala nuclei in patient groups.

A

	SCZ-V vs SCZ-NV			
Region	*B-coefficient*	*SE*	*p-value*	*Effect size*
whole hippocampus	-34.5	182.5	0.927	-0.059
parasubiculum	-7.6	6.3	0.927	-0.384
presubiculum	-20.4	21.2	0.927	-0.305
subiculum	-44.2	73	0.927	-0.191
CA1	-6.7	38.8	0.927	-0.054
CA3	28.1	15.3	0.927	0.581
CA4	13.7	14.2	0.927	0.305
GCMLDG	13.3	16.3	0.927	0.259
HATA	-5.2	4.9	0.927	-0.338
fimbria	-12.3	10.4	0.927	-0.373
molecular layer	4.2	29.3	0.927	0.0458
hippocampal fissure	15	13.8	0.927	0.345
hippocampal tail	-53.5	52	0.927	-0.326
whole amygdala	20	93.7	0.927	0.067
lateral nucleus	8.3	38.7	0.927	0.068
basal nucleus	5.9	40.4	0.927	0.046
accessory basal nucleus	2.3	16.3	0.927	0.045
anterior amygdaloid area	2.7	4.7	0.927	0.184
central nucleus	1.3	4	0.927	0.107
medial nucleus	2.5	2.8	0.927	0.278
cortical nucleus	1.4	2	0.927	0.217
corticoamygdaloid transition area	-2.2	10.6	0.927	-0.067
paralaminar nucleus	0.1	2.8	0.979	0.008

B

	SCZ-V vs SCZ-NV			
Region	*B-coefficient*	*SE*	*p-value*	*Effect size*
whole hippocampus	-59.8	178.7	0.904	-0.105
parasubiculum	-5.6	6.1	0.75	-0.293
presubiculum	-27.4	20.8	0.75	-0.415

subiculum	-75.3	71.4	0.75	-0.333
CA1	-16.9	39.3	0.9045	-0.136
CA3	28.5	15.8	0.582	0.569
CA4	15.8	14.1	0.75	0.353
GCMLDG	15.1	16.4	0.75	0.292
HATA	-9	4.8	0.582	-0.590
fimbria	-14.8	10.3	0.75	-0.455
molecular layer	-6.4	28.9	0.904	-0.069
hippocampal fissure	29.8	14.1	0.582	0.667
hippocampal tail	-29.7	49.9	0.904	-0.188
whole amygdala	-13.3	95.1	0.929	-0.044
lateral nucleus	1.6	37.9	0.966	0.013
basal nucleus	-13	42.3	0.904	-0.097
accessory basal nucleus	-4	16.9	0.904	-0.075
anterior amygdaloid area	1.4	4.6	0.904	0.097
central nucleus	0.9	3.9	0.904	0.074
medial nucleus	3.7	2.8	0.75	0.412
cortical nucleus	2.2	1.9	0.75	0.359
corticoamygdaloid transition area	-8.7	10.5	0.787	-0.261
paralaminar nucleus	-1.4	2.7	0.904	-0.165

C

	**SCZ-V vs SCZ-NV**			
Region	*B-coefficient*	*SE*	*p-value*	*Effect size*
whole hippocampus	-159.2	188.2	0.768	-0.269
parasubiculum	-8.7	6.1	0.601	-0.456
presubiculum	-44.8	21.4	0.601	-0.668
subiculum	-129.7	73.9	0.601	-0.559
CA1	-29.4	40.5	0.782	-0.231
CA3	27.4	15.6	0.601	0.560
CA4	5.4	14.5	0.909	0.119
GCMLDG	6.4	16.5	0.909	0.124
HATA	-4.7	4.8	0.768	-0.307

fimbria	-16.2	10.5	0.601	-0.491
molecular layer	-21.7	30.3	0.782	-0.228
hippocampal fissure	16.3	14.5	0.768	0.357
hippocampal tail	-50.3	51.2	0.768	-0.313
whole amygdala	-18.8	94.9	0.946	-0.063
lateral nucleus	-12.3	38.8	0.909	-0.101
basal nucleus	-13.2	41	0.909	-0.102
accessory basal nucleus	-2	16.7	0.947	-0.037
anterior amygdaloid area	-0.1	4.6	0.982	-0.007
central nucleus	3.4	4	0.768	0.271
medial nucleus	4.4	3	0.601	0.461
cortical nucleus	2.1	2	0.768	0.333
corticoamygdaloid transition area	-1.8	10.6	0.946	-0.054
paralaminar nucleus	-1.2	2.8	0.909	-0.134

a Regression analysis on hippocampal subfields and amygdala nuclei between SCZ-V and SCZ-NV covaried for antipsychotic medication use **b** Regression analysis on hippocampal subfields and amygdala nuclei between SCZ-V and SCZ-NV covaried for duration of illness **c** Regression analysis on hippocampal subfields and amygdala nuclei between SCZ-V and SCZ-NV covaried for illicit substance use

Abbreviations: SCZ-V schizophrenia patients with a history of violence, SCZ-NV schizophrenia patients with no history of violence, HC healthy controls, CA cornu ammonis, GCMLDG granule cell layer of dentate gyrus, HATA hippocampal-amygdaloid transition area

All analyses were covaried for age, age, age^2, sex, intracranial volume (ICV) and scanning site.
All p-values are FDR-corrected for multiple comparisons.

Hippocampal subfield and amygdala nuclei volumes in schizophrenia patients with a history of violence; European Archives of Psychiatry and Clinical Neuroscience; Natalia Tesli, Dennis van der Meer, Jaroslav Rokicki, Guttorm Storvestre, Cato Røsæg, Arvid Jensen, Gabriela Hjell, Christina Bell, Thomas Fischer-Vieler, Martin Tesli, Ole A Andreassen, Ingrid Melle, Ingrid Agartz, Unn K Haukvik*
*Corresponding author: Haukvik UK, MD, Ph.D, email: u.k.h.haukvik@medisin.uio.no, postal address: Oslo University Hospital, P.O.Box 4956 Nydalen, 0424 Oslo, Norway, phone: +47 23 02 73 50
