# Supplementary material for: Hippocampal subfield and amygdala nuclei volumes in schizophrenia patients with a history of violence
Source: Eur Arch Psychiatry Clin Neurosci. 2020 Jan 24;270(6):771–82. doi: 10.1007/s00406-020-01098-y (PMC7423802; doi:10.1007/s00406-020-01098-y)
Supplement: Supplementary file 2 — Supplementary file2 (PDF 255 kb) [file 406_2020_1098_MOESM2_ESM.pdf]

**Supplementary Fig. S1** Violin plots showing distributions of the original brain volumes in schizophrenia patients with a history of violence, schizophrenia without a history of violence and healthy controls.

A

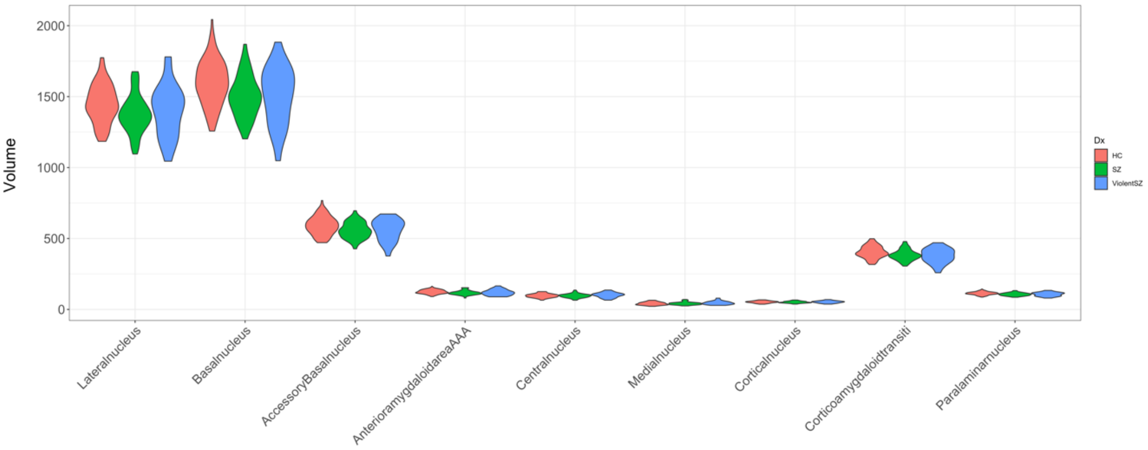


B

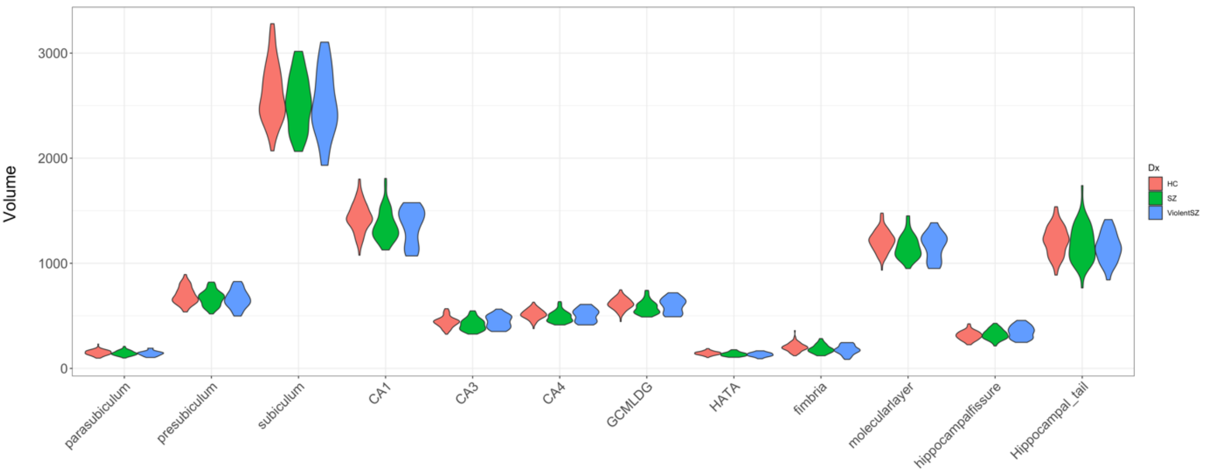


**a** Volumetric distribution of amygdala nuclei in SCZ-V, SCZ-NV and HC **b** Volumetric distribution of hippocampal subfields in SCZ-V, SCZ-NV and HC. Results are uncorrected for covariates.

Abbreviations: Dx diagnosis, violent SCZ schizophrenia patients with a history of violence, SCZ schizophrenia patients without a history of violence, HC healthy controls, CA cornu ammonis, GCMLDG granule cell layer of dentate gyrus, HATA hippocampal-amygdaloid transition area

Hippocampal subfield and amygdala nuclei volumes in schizophrenia patients with a history of violence; European Archives of Psychiatry and Clinical Neuroscience; Natalia Tesli, Dennis van der Meer, Jaroslav Rokicki, Guttorm Storvestre, Cato Røsæg, Arvid Jensen, Gabriela Hjell, Christina Bell, Thomas Fischer-Vieler, Martin Tesli, Ole A Andreassen, Ingrid Melle, Ingrid Agartz, Unn K Haukvik*
*Corresponding author: Haukvik UK, MD, Ph.D, email: u.k.h.haukvik@medisin.uio.no, postal address: Oslo University Hospital, P.O.Box 4956 Nydalen, 0424 Oslo, Norway, phone: +47 23 02 73 50
